# Periodontitis and Preeclampsia in Pregnancy: A Systematic Review and Meta-Analysis

**DOI:** 10.1007/s10995-022-03556-6

**Published:** 2022-10-08

**Authors:** Quynh-Anh Le, Rahena Akhter, Kimberly Mathieu Coulton, Ngoc Truong Nhu Vo, Le Thi Yen Duong, Hoang Viet Nong, Albert Yaacoub, George Condous, Joerg Eberhard, Ralph Nanan

**Affiliations:** 1 School of Dentistry and the Charles Perkins Center, Faculty of Medicine and Health, The University of Sydney, Sydney, NSW Australia; 2 Department of Pediatric Dentistry, School of Odonto-Stomatology, Hanoi Medical University, Hanoi, Vietnam; 3 Present Address: School of Odonto-Stomatology, Hanoi Medical University, Hanoi, Vietnam; 4 Nepean Centre for Oral Health, Nepean Hospital, Kingswood, NSW Australia; 5 Acute Gynaecology, Early Pregnancy, and Advanced Endoscopy Surgery Unit, Sydney Medical School Nepean, Nepean Hospital, University of Sydney, Sydney, Australia; 6 Sydney Medical School Nepean and Charles Perkins Center Nepean, The University of Sydney, Sydney, NSW Australia

**Keywords:** Periodontitis, Periodontal disease, Preeclampsia, Pre-eclampsia, Hypertension, Pregnancy outcome

## Abstract

**Objectives:**

A conflicting body of evidence suggests localized periodontal inflammation spreads systemically during pregnancy inducing adverse pregnancy outcomes. This systematic review and meta-analysis aim to specifically evaluate the relationship between periodontitis and preeclampsia.

**Methods:**

Electronic searches were carried out in Medline, Pubmed, Embase, Lilacs, Cochrane Controlled Clinical Trial Register, CINAHL, ClinicalTrials.gov, and Google Scholar with no restrictions on the year of publication. We identified and selected observational case–control and cohort studies that analyzed the association between periodontal disease and preeclampsia. This meta-analysis was conducted following the PRISMA checklist and MOOSE checklist. Pooled odds ratios, mean difference, and 95% confidence intervals were calculated using the random effect model. Heterogeneity was tested with Cochran’s Q statistic.

**Results:**

Thirty studies including six cohort- and twenty-four case–control studies were selected. Periodontitis was significantly associated with increased risk for preeclampsia (OR 3.18, 95% CI 2.26 – 4.48, p < 0.00001), especially in a subgroup analysis including cohort studies (OR 4.19, 95% CI 2.23 – 7.87, p < 0.00001). The association was even stronger in a subgroup analysis with lower-middle-income countries (OR 6.70, 95% CI 2.61 – 17.19, p < 0.0001).

**Conclusions:**

Periodontitis appears as a significant risk factor for preeclampsia, which might be even more pronounced in lower-middle-income countries. Future studies to investigate if maternal amelioration of periodontitis prevents preeclampsia might be warranted.

## Significance

The most recent systematic review and meta-analysis on the relationship between periodontal disease and preeclampsia were published in 2013 and could only detect the statistical differences in a subgroup of case-control studies. For that reason, we conducted this study to re-evaluate this association, especially with regard to cohort study design. Furthermore, we take socioeconomic factors into consideration. This systematic review and meta-analysis not only empower the positive association between periodontitis and preeclampsia during pregnancy by the larger sample size and the data synthesis of cohort studies but also point out the considerable difference in lower-middle-income countries.

## Introduction

Preeclampsia is the onset of pregnancy-related hypertensive disorder and proteinuria arising most commonly after 20 weeks of gestation, which could lead to eclampsia and induce maternal and perinatal morbidity, and mortality. The prevalence of preeclampsia is between 2 to 8% of all pregnancies worldwide (Duley, 2009). Preeclampsia affected pregnancies had a higher risk of poor maternal outcomes including cerebrovascular bleeding, HELLP syndrome, eclampsia, poorer outcomes of their offspring including premature birth, intrauterine growth restriction, and the complications may manifest over years postpartum (Hung et al., 2018; Turbeville & Sasser, 2020).

Contributing to USD 6.4 billion short-term estimated costs for preeclamptic pregnancies in US healthcare system, USD 1.03 billion were spent on maternal healthcare and USD 1.15 billion were expended for infants born to these women while the remaining expenses were for peripartum and postpartum care (Stevens et al., 2017).

Herein, managing risk factors of preeclampsia is important to improve maternal and perinatal outcomes and lessen the burden on the health economic aspects.

Depending on geographical regions approximately 14.2 and 54.8% of pregnant women suffer from periodontal disease (Alchalabi et al., 2013; Gesase et al., 2018; Govindasamy et al., 2017). Especially, periodontitis, a more severe type of periodontal diseases affecting 11% of the pregnant women, can cause the destruction of periodontal tissue and cause systemic dissemination of bacteria and other inflammatory mediators (Bui et al., 2019; Piscoya et al., 2012). Systemic inflammatory processes triggered by focal periodontal infections have been attributed to cardiovascular, cerebrovascular diseases and respiratory diseases.(Winning & Linden, 2015) Periodontitis has independently been linked to several pregnancy complications such as preterm birth, low birth weight, and gestational diabetes (Abariga & Whitcomb, 2016; Corbella et al., 2012).

Socioeconomic status is a recognized factor associated with medical outcomes, including pregnancy outcomes (Kivimäki et al., 2020). Women with lower socioeconomic status are at a higher risk of pregnancy complications such as gestational diabetes, preterm delivery, and preeclampsia (Bo et al., 2002; Peacock et al., 1995; Silva et al., 2008). Women with high socioeconomic status have a statistically significant reduced risk of preeclampsia with an odds ratio of 0.899 (95% CI, 0.862–0.937, p < 0.001) compared to women with lower socioeconomic statuses (Ross et al., 2019). At the same time, the proportion of periodontitis in pregnancy is linked to low socioeconomic status with 42.6%, compared to high socioeconomic status with 15.0%.

Two previous meta-analyses both published in 2013 reported positive associations between preeclampsia and periodontitis with OR 2.17, 95% CI 1.38–3.41, p = 0.008 and OR of 2.79, 95% CI 2.01–3.01, p < 0.0001, but did not consider socioeconomic factors (Sgolastra et al., 2013; Wei et al., 2013). Since then further case–control and cohort studies have been published on this research topic (Lafaurie et al., 2018; Soucy-Giguère et al., 2016; Varshney & Gautam, 2014). Nonetheless, the causal relationship between periodontal disease and preeclampsia remains unclear (Kunnen et al., 2010; Lavigne & Forrest, 2020). In this review, we included all available new studies, to re-evaluate the potential association between periodontitis and preeclampsia and also to take socioeconomic factors into consideration (Australian Institute of Health and Welfare 2010. Socioeconomic variation in periodontitis among Australian adults 2004–06.)

## Methods

### Eligibility Criteria

The studies were screened according to the following inclusion criteria:

1. Study design was either case–control or prospective cohort study;
2. Studies analysing the association between periodontal disease and preeclampsia;
3. Study population was pregnant women without systemic diseases;
4. Preeclampsia was defined as the development of blood pressure of ≥ 140/90 mmHg after 20 weeks of gestation, combined with proteinuria of at least 1 + on midstream urine specimen or catheter specimen;
5. Periodontitis was either diagnosed ≥ 2 sites with PD ≥ 4 mm and CAL ≥ 3 mm, not on the same site or one site with PD ≥ 5 mm at the same site or evaluated the progression by clinical periodontal parameters including periodontal pocket depth, clinical attachment loss and bleeding on probing (Eke et al., 2012). The progression was measured by pocket depth (mm), clinical attachment level (mm) at baseline and delivery time;
6. Data was presented in such a way that Odds Ratio and 95% Confidence Interval could be calculated.

Studies were excluded if they did not report adequate data on periodontal or preeclamptic conditions or outcome of interest or did not meet the inclusion criteria.

### Information Sources

We followed the Preferred Reporting Items for Systematic Reviews and Meta-Analysis (PRISMA) guidelines with the checklist of 27 items to conduct our study (Moher et al., 2009). We also adopted the MOOSE checklist for Meta-analysis of Observational studies (Stroup et al., 2000). A systematic search of the electronic database including Medline (from 1950), Pubmed (from 1946), Embase (from 1949), Lilacs, Cochrane Controlled Clinical Trial Register, CINAHL, ClinicalTrials.gov and Google Scholar (from 1990) to identify relevant articles.

### Search Strategy

We used the following search terms: periodontitis, periodontal disease, preeclampsia, pre-eclampsia, pregnancy outcomes, pregnancy complications, and hypertension. The combinations of search terms were used to explore above databases. The search strategy was peer reviewed by two independent reviewers (QA and LD). The reference lists of relevant articles were also scanned for appropriate studies. No language restrictions were adopted in either the search or study selection. No search for unpublished literature was carried out. Authors were contacted for translation and information.

### Study Selection

Two independent reviewers (LD and HN) reviewed the titles, abstracts and methods of retrieved results to assess for the eligibility criteria. When there was a disagreement in a selection process between reviewers, consensus with the third reviewer (QA) was obtained.

### Data Extraction

Data extraction was carried out using a standardized extraction form, collecting information on the first author’s name, publication year, study design, number of cases, number of controls, total sample size, country, national income group, mean age, the risk of estimates or data used to calculate the risk estimates, CIs or data used to generate CI. According to the World Bank classification of countries which is based on Gross National Income per capita, groups of national income per year are (according to World Bank classification (Country and Lending Groups & The World Bank Group, 2011)):

- Low income: $995 or less;
- Lower-middle-income: $996–$3945;
- Upper-middle-income: $3946–$12,195;
- High income: $12,196 or more.

The researchers cross-checked all extracted data and discussed if there were disagreements.

### Assessment of Risk of Bias

Risk of bias was executed using the Newcastle Ottawa Scale by two reviewers (QA and LD) (Lo et al., 2014) with disagreements resolved by consensus attainment between reviewers. This scale has three components including Selection, Comparability and Outcome/Exposure assessment with maximum overall score of nine. Studies were rated as low risk of bias if they received nine score, moderate risk of bias if they received seven or eight score and high risk of bias if they received less than seven scores.

### Data Synthesis

Data were imported in a statistical software (RevMan, Version 5, 2008, The Nordic Cochrane Center, The Cochrane Collaboration, Copenhagen, Denmark). Pooled Odds Ratios, mean difference, and 95% Confidence Intervals were calculated for the association between periodontitis and preeclampsia using a random effects model. The pooled effect was considered significant if p-value was less than 0.05. Forest plots for primary analysis and subgroup analysis show the raw data, Odds Ratio and CIs, Means and SDs for the chosen effect, heterogeneity statistic (I^2^), total number of participants per group, overall Odds Ratio and Mean difference.

Subgroup analysis was carried out according to the study design (case–control or cohort), definition of periodontitis (defined by pocket depth (PD) and/or clinical attachment loss (Taghzouti et al.)), mean CAL, mean PD, national income (high-income or middle-income or low-income countries).

Heterogeneity was tested with Cochran’s Q statistic, with p < 0.10 indicating heterogeneity, and quantified the degree of heterogeneity using the I^2^ statistic, which represents the percentage of the total variability across studies which is due to heterogeneity. I^2^ values of 25, 50 and 75% corresponded to low, moderate and high degrees of heterogeneity respectively (Higgins & Thompson, 2002). We quantified publication bias using the Egger’s regression model with the effect of bias assessed using the fail-safe number method (Egger et al., 1997) The fail-safe number was the number of studies that we would need to have missed for our observed result to be nullified to statistical non-significance at the p < 0.05 level. Publication bias is generally regarded as a concern if the fail-safe number is less than 5n + 10, with n being the number of studies included in the meta-analysis (Orwin, 1983) Publication bias was assessed using Stata (16.1, StataCorp LLC, College Station, TX).

## Results

### Study Selection

**Fig. 1:**
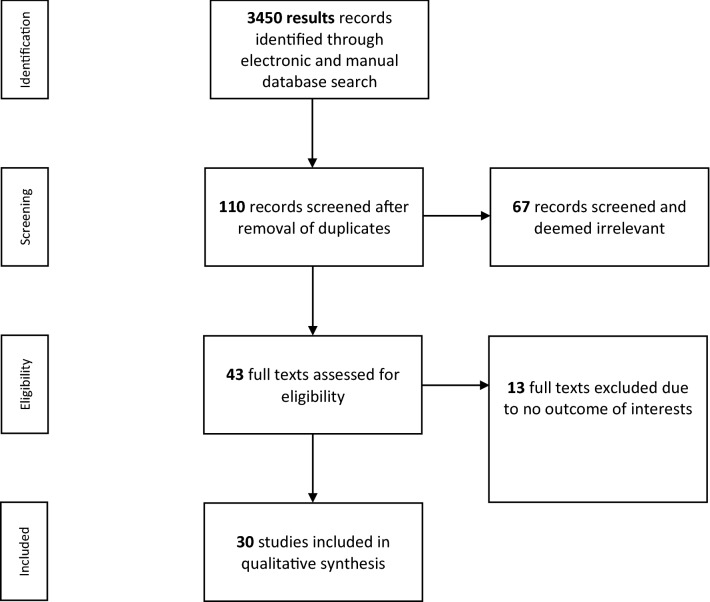
Flow diagram of study selection

A total of 3450 articles were found through the manual and electronic searches. We searched clinicaltrials.gov however, we could not find eligible articles from this source to include in our study. After duplicates’ removal, we screened 110 records for relevance. Sixty-seven papers were excluded on a basis of evaluation of the title and abstract, leaving 43 articles to be assessed for eligibility. Of these, thirty articles were included in the quantitative analysis. A PRISMA flow diagram is provided in Fig. 1. Eventually, the selection process led to the inclusion of 9650 participants in this systematic review and meta-analysis.

### Study Characteristics

Table 1 depicts the characteristics of the included studies. Six cohort studies were included (Boggess et al., 2003; Ha et al., 2014; Horton et al., 2010; Kumar et al., 2013; Lee et al., 2016; Soucy-Giguère et al., 2016), whilst the remaining studies were case – control (Canakci et al., 2004, 2007; Chaparro et al., 2013; Contreras et al., 2006; Cota et al., 2006; Desai et al., 2015; Ha et al., 2011; Hirano et al., 2012; Jaiman et al., 2018; Khader et al., 2006; Khalighinejad et al., 2017; Kunnen et al., 2007; Lafaurie et al., 2018; Lohsoonthorn et al., 2009; Moura da Silva et al., 2012; Pattanashetti et al., 2013; Politano et al., 2011; Pralhad et al., 2013; Sayar et al., 2011; Shetty et al., 2009; Siqueira et al., 2008; Taghzouti et al., 2012; Varshney & Gautam, 2014; Yaghini et al., 2012). The definitions and evaluation of periodontitis varied slightly among these studies with the assessment of bleeding on probing, while the definition of preeclampsia was presented consistent as blood pressure ≥ 140/90 mmHg and proteinuria during second trimester of gestation. The oral examination was conducted at different timepoints among studies, within two days of childbirth(Boggess et al., 2003; Canakci et al., 2004, 2007; Cota et al., 2006; Desai et al., 2015; Ha et al., 2014; Horton et al., 2010; Jaiman et al., 2018; Khader et al., 2006; Khalighinejad et al., 2017; Kumar et al., 2013; Lohsoonthorn et al., 2009; Moura da Silva et al., 2012; Pattanashetti et al., 2013; Politano et al., 2011; Pralhad et al., 2013; Sayar et al., 2011; Shetty et al., 2009; Siqueira et al., 2008; Taghzouti et al., 2012; Varshney & Gautam, 2014; Yaghini et al., 2012), within 7 days after the delivery(Ha et al., 2011; Hirano et al., 2012; Lafaurie et al., 2018), 3 months postpartum(Kunnen et al., 2007), during the second trimester of pregnancy(Chaparro et al., 2013; Contreras et al., 2006; Lee et al., 2016; Soucy-Giguère et al., 2016). Nineteen out of thirty studies reported the implementation of calibration with the intra- and inter-examiner variability which showed the agreement of 85% and above(Boggess et al., 2003; Canakci et al., 2004, 2007; Cota et al., 2006; Ha et al., 2011, 2014; Horton et al., 2010; Jaiman et al., 2018; Khalighinejad et al., 2017; Kunnen et al., 2010; Lafaurie et al., 2018; Lee et al., 2016; Lohsoonthorn et al., 2009; Moura da Silva et al., 2012; Politano et al., 2011; Sayar et al., 2011; Siqueira et al., 2008; Taghzouti et al., 2012).

**Table 1:** Descriptions of included studies

No	References	Country	Design	Participants and age	Definition of PE	Definition of PD	Examination Time	Finding (conclusion)	OR (95% CI)
1	(Boggess et al., 2003)	US	Prospective cohort	39 exposed 763 unexposed	BP ≥ 140/90 on 2 separate occasions, and ≥ 1 + proteinuria on catheterized urine specimen	PD ≥ 4 and CAL ≥ 3 mm without BOP Mild: PD ≥ 4 mm or BOP on 1–15 teeth Severe: PD ≥ 4 mm on > 15 teeth Disease progression: ≥ 4 sites that increased ≥ 2 mm in PD, resulting in ≥ 4 mm in PD	At the first or second prenatal visit and then repeated within 48 h antepartum Enrolled at < 26 weeks’gestation and followed until delivery	Women were at higher risk for preeclampsia if they had severe periodontal disease at delivery or if they had periodontal disease progression during pregnancy	Severe periodontal disease: 2.4 (1.1- 5.3) Periodontal disease progression: 2.1 (1.0 – 4.4)
2	(Canakci et al., 2004)	Turkey	Case–control	41 cases,41 controls	BP ≥ 140/90 mmHg and proteinuria ≥ 300 mg/24 h or 2 + proteinuria on dip sticks, on 2 occasions ≥ 6 h apart if 24 h urine specimen is unavailable	≥ 4 teeth with ≥ 1 sites with PD ≥ 4 mm and BOP + and CAL ≥ 3 mm at the same site	within 48 h prior to delivery	multiple logistic regression results showed that pre-eclamptic patients were 3.47 times more likely to have periodontal disease than normotensive patients	3.47 (1.07–11.95)
3	(Contreras et al., 2006)	Colombia	Case–control	130 case, 243 controls	2 + proteinuria, confirmed by ≥ 0.3 g proteinuria/24 h and hypertension (≥ 140/ 90 mmHg)	≥ 4 sites showed PD ≥ 4 mm), CAL ≥ 4 mm, and bleeding on probing Incipient: CAL from 4 to 5 Moderate/severe: CAL ≥ 6 mm	between 26 to 36 weeks of pregnancy	Chronic periodontal disease was significantly associated with preeclampsia in pregnant women	3.0 (1.91—4.87)
4	(Cota et al., 2006)	Brazil	Case–control	109 cases, 479 controls	BP > 140/90 mm Hg and ≥ 1 + proteinuria after 20 weeks of gestation	≥ 4 teeth with ≥ 1 sites with a PD ≥ 4 mm and CAL ≥ 3 mm at the same site	Within 48 h of delivery	Maternal periodontitis was determined to be associated with an increased risk of preeclampsia	1.88 (1.1—3.0)
5	(Khader et al., 2006)	Jordan	Case–control	115 cases 230 controls	Preeclampsia was defined as the development of blood pressure of ‡140/90 mmHg after 20 weeks of gestation, combined with proteinurea of at least 1 + on a midstream urine specimen or on a catheter specimen, provided urinary tract infection was not the contributing factor to the proteinuria in women who were known to be normotensive and nonproteinuric before pregnancy or in early pregnancy	Not mentioned	Within 24 h after delivery	This study did not support the association between periodontal parameters and preeclampsia	
6	(Kunnen et al., 2007)	Netherlands	Case–control	17 cases, 35 controls	DBP ≥ 90 mmHg on 2 occasions and proteinuria ≥ 30 mg/dl (or 1 + on a urine dip stick) on ≥ 2 random specimens collected ≥ 4 h apart	PD ≥ 4 mm Mild PD: BOP and PD ≥ 4 mm on 1–15 sites Severe: BOP and PD ≥ 4 mm on > 15 sites	before 34 weeks of pregnancy and 3 months postpartum	These results indicate that Caucasian women with a recent history of early-onset pre-eclampsia have a worse periodontal condition, as compared with women with uncomplicated deliveries	7.9 (1.9–32.8)
7	(Canakci et al., 2007)	Turkey	Case–control	20 Mild PE, 18 Severe PE, 21 Controls	DBP ≥ 90 mmHg and proteinuria(300 mg/24 h urine sample) and the presence of edema Mild: BP ≥ 140/90 mmHg on ≥ 2 occasions 6 h apart, with or without proteinuria Severe: SBP ≥ 160 or DBP ≥ 110 mmHg on 2 occasions ≥ 6 h apart and proteinuria ≥ 5 g/24 h urine sample or ≥ 3 l on dip stick in ≥ 2 random clean-catch samples ≥ 4 h apart	Mild: BOP and ≥ 4 mm PD on 1–15 sites Severe:: BOP and ≥ 4 mm PD on ≥ 15sites	within 48 h preceding delivery	The results of multivariate logistic regression showed a highly significant association between mild to severe pre-eclampsia and severe periodontal disease	Severe PD: 3.78 (1.77–12.74)
8	(Siqueira et al., 2008)	Brazil	Case–control	164 cases, 1042 controls	BP > 90 mmHg on 2 occasions after 20 GW and ≥ 1 + proteinuria	≥ 4 mm and CAL ≥ 3 mm at the same site in ≥ 4 teeth	within 48 h of delivery	Maternal periodontitis is a risk factor associated with preeclampsia	1.52 ( 1.01—2.29)
9	(Lohsoonthorn et al., 2009)	Thailand	Case–control	150 cases, 150 controls	BP ≥ 140/90 mmHg and proteinuria ≥ 30 mg/dl (or 1 + on a urine dip stick) on ≥ 2 random specimens collected ≥ 4 h apart	Mild: ≥ 1 teeth with interproximal sites showing ≥ 4 mm CAL and ≥ 4 mm PD Moderate: ≥ 2 nonadjacent teeth with interproximal sites showing ≥ 5 mm CAL and ≥ 4 mm PD Severe: ≥ 2 nonadjacent teeth with interproximal sites showing ≥ 6 mm CAL and ≥ 4 mm PD	within 48 h after delivery	This study provides no convincing evidence that periodontal disease is associated with preeclampsia risk among Thai women	Severe PD: 0.92 ( 0.26–3.28)
10	(Horton et al., 2010)	US	Prospective cohort	34 exposed(pree-clampsia) 757 unexposed (non pree-clampsia)	BP > 140/90 mmHg and ≥ 1 + proteinuria on a catheterized urine specimen	Mild: < 15 sites with the presence of one or more pockets ≥ 4 mm or one or more pockets with bleeding Moderate/severe: ≥ 15 sites demonstrated a probing depth ≥ 4 mm	< 26 weeks of gestation	Among women with periodontal disease, the presence of 8-isoprostane ≥ 75th percentile did not significantly increase the odds for the development of preeclampsia	2.08 (0.65–6.60)
11	(Shetty et al., 2009)	India	Case–control	30 cases 100 controls	BP > 140/90 mmHg on more than 2 occasions 4 h apart and 1 + or more proteinuria by Dipstick on a random urine sample	CAL of ≥ 3 mm and a PD of ≥ 4 mm (The teeth examined were 16, 22, 24, 36, 42, and 44)	within 48 h of delivery	periodontitis both at enrolment and within 48 h of delivery may be associated with an increased risk of preeclampsia	Enrolment:5.78 (2.41– 13.89) Delivery: 20.15 (4.55–89.29)
12	(Politano et al., 2011)	Brazil	Case–control	58 cases 58 controls	increase in systolic arterial pressure (≥ 140 mmHg) and/or diastolic pressure (≥ 90 mmHg) and proteinuria (≥ 300 mg/24 h), after 20 wk of gestation	two or more sites showed pocket formation (≥ 4 mm), clinical attachment level (≥ 4 mm) and bleeding on probing	after 20 wk of gestation	periodontal disease may increase the risk of pre-eclampsia	3.73 (1.32–10.58)
13	(Ha et al., 2011)	Korea	Case–control	16 cases 48 controls	BP > 140/90 mm Hg on two separate occasions and ≥ 1 + proteinuria on a random sample of urine	Localized periodontitis: periodontal clinical attachment loss ≥ 3.5 mm on two or three sites not on the same tooth Generalized periodontitis: CAL ≥ 3.5 mm on ≥ 4 sites not on the same tooth	5 days after delivery	preeclampsia could be associated with the maternal periodontal condition and interdental cleaning	Localized periodontitis: 4.79 (1.02 – 29.72) Generalized periodontitis: 6.60 (1.25 –41.61)
14	(Sayar et al., 2011)	Iran	Case–control	105 cases 105 controls	blood pressure ≥ 140/90 mmHg and proteinuria + 1	No definition specified (results based on periodontal parameters) Mild: CAL ≤ 2 mm Moderate to Severe: CAL ≥ 3 mm	48 h after child delivery	Preeclamptic cases significantly had higher attachment loss and gingival recession than the control group	4.1 (1.5–11.5)
15	(Taghzouti et al., 2012)	Canada	Case–control	92 cases 245 controls	BP ≥ 140/90 mm Hg on two occasions ≥ 4 h apart after 20 weeks of gestation, and 0.3 g proteinuria on a 24-h urine collection, or ≥ 1 on a dipstick	periodontitis is defined as ≥ 4 sites exhibiting PD ≥ 5 mm and CAL ≥ 3 mm at the same sites	within 48 h after delivery	This study does not support the hypothesis of an association between periodontal disease and preeclampsia	1.13 (0.59—2.17)
16	(Chaparro et al., 2013)	Chile	Case–control	11 cases 43 controls	During the second and third trimester of pregnancy, BP > 140/90 and proteinuria, which was considered to be present when one 24-h urine collection showed a total protein excretion ≥ 300 mg	PD ≥ 4 mm and CAL ≥ 3 mm at the same site of ≥ 4 teeth, inflammation and bleeding on probing (BOP)	Blood samples were collected at enrolment Gingival crevicular fluid samples were collected between 11–14 wk	Preeclamptic women shows increased levels of IL-6 in GCF and CRP in plasma during early pregnancy. Periodontal disease could contribute to systemic inflammation in early pregnancy via a local increase of IL-6 and the systemic elevation of CRP. Therefore, both inflammatory markers could be involved in the relationship between periodontal disease and pre-eclampsia	1.36 (0.252–7.372)
17	(Hirano et al., 2012)	Japan	Case–control	18 cases 109 controls	hypertension (systolic blood pressure > 140 mmHg and/or diastolic blood pressure > 90 mmHg) with proteinuria ( 300 mg/day) occurring after the 20th week of gestation, but being resolved by the 12th postpartum week	having over 60% of sites with CAL ≥ 3 mm	5 days after labor	No statistically significant association between any of the periodontal clinical parameters or the presence of periodontitis and preeclampsia	1.7 (1.1–2.7)
18	(Kumar et al., 2013)	India	Prospective cohort	35 exposed 305 unexposed	systolic blood pressure 140 mm of mercury and diastolic blood pressure 90 mm of mercury at two occasions at least 4 h apart after 20 weeks of gestation in a woman with previously normal blood pressure along with development of proteinuria	clinical attachment loss and probing depth 4 ≥ mm in one or more sites were diagnosed as those with periodontitis	14–20 weeks period of gestation and the time of delivery	Maternal periodontitis is associated with an increased risk of pre-eclampsia	7.48 (2.72–22.42)
19	Da (Silva et al., 2008)	Brazil	Case–control	284 cases 290 controls	a systolic blood pressure ≥ 140 mmHg or a diastolic pressure ≥ 90 mmHg and proteinuria ≥ 300 mg/24 h or 2 + on dipsticks, developed after week 20 of gestation in previously normotensive females	≥ 4 teeth with ≥ 1 sites with a PD ≥ 4 mm and AL ≥ 3 mm in the same site	within 48 h of childbirth	periodontitis was a risk factor for preeclampsia	8.60 ( 3.92–18.88)
20	(Pralhad et al., 2013)	India	Case–control	100 cases 100 controls	The resting blood pressure was ≥ 140/90 mmHg after 20 weeks of gestation with or without associated proteinuria	Any of the following is present: 1) OHI > 3 (Hosseinpoor et al.); 2) GI > 1 (moderate-to-severe gingival inflammation); 3) mean PD > 4 mm; and 4) CAL > 3 mm	within 72 h of their hospital admission for delivery	Periodontal disease is more prevalent in females with pregnancy hypertension	5.5 (2.7–11.4)
21	(Yaghini et al., 2012)	Iran	Case–control	26 cases 25 controls	blood pressure > 140/90 mmHg and > or = 1 + proteinuria on a catheterized urine specimen	Periodontal assessment was carried out using these indices: Clinical attachment loss, Gingival bleeding index, plaque index	48 h after delivery	Maternal periodontal disease during pregnancy is not associated with preeclampsia	
22	(Pattanashetti et al., 2013)	India	Case–control	100 cases 100 controls	Pregnancy induced hypertension occurs after the 20th week of gestation and is characterized by: Hypertension – High blood pressure, usually higher than 140/90 mm Hg. The rise of blood pressure should be evident at least on two occasions, four or more hours apart Edema – Demonstration of pitting oedema over the ankles after 12 h of bed rest or rapid gain in weight of more than 1 lb per week or more than 5 lb a month in the later month of pregnancy may be the easiest evidence of preeclampsia Proteinuria – Presence of protein in 24 h urine with more than 1gm per litre in 2 or more midstream specimens obtained 6 h apart in the absence of urinary tract infection is considered significant	-Mild periodontal disease: One or more sites with probing depth ≥ 3 mm that bleed upon probing but less than 25 sites with probing depth ≥ 4 mm -Moderate/Severe: 15 or more sites with periodontal probing ≥ 4 mm -Worsening periodontal status was defined as four or more sites had increased by at least 2 mm in pocket depth between the two oral health examinations	Sixth month of pregnancy and within 48 h post-partum	Pregnant women with preeclampsia are at greater risk for preterm delivery if periodontal disease is present during pregnancy or progress during pregnancy and also the rate of preterm delivery is more in preeclamptic women having moderate to severe periodontal disease	Periodontitis in the case group was 72%, in control group was 62%, p < 0.001
23	(Ha et al., 2014)	Korea	Prospective cohort	13 exposed 270 unexposed	BP > 140/90 mmHg on two separate occasions, and at least 1 + proteinuria on a random urine screen after the 20th week of pregnancy	CAL ≥ 4.0 mm on two or more sites on different teeth	21–24 weeks of gestation and 5 days after delivery	Periodontitis increased the risk of preeclampsia among never-smokers	4.51 (1.13–17.96)
24	(Varshney & Gautam, 2014)	India	Case–control	20 cases 20 controls	BP ≥ 140/90 mm of Hg on two separate occasions after 20 week of gestation and ≥ 1 + proteinuria	PD ≥ 4 mm and CAL ≥ 3 mm at the same site on at least 4 different non-neighboring teeth	within the 48 h after delivery	Maternal clinical periodontal disease at delivery is associated with an increased risk for the development of pre-eclampsia, independent of the effects of maternal age, race, smoking, gestational age at delivery	4.33 (1.15- 16.32)
25	(Desai et al., 2015)	India	Case–control	120 cases 1120 controls	Blood pressure ≥ 140/90 mm Hg on two separate occasions after week 20 of gestation	PD ≥ 4 mm and CAL ≥ 3 mm at the same site in at least four teeth	48 h after delivery	Maternal clinical periodontal disease at delivery is associated with an increased risk for the development of pre-eclampsia, independent of the effects of maternal age, race, smoking, gestational age at delivery	19.898 (7.80–48.94)
26	(Soucy-Giguère et al., 2016)	Canada	Prospective cohort	11 exposed 237 unexposed	Not mentioned	the presence of at least one site with probing depths ≥ 4 mm and ≥ 10% bleeding on probing	in the seven days following amniocentesis	Pregnant women with periodontal disease were more likely to develop preeclampsia	RR 5.89 (1.24–28.05)
27	(Lee et al., 2016)	Korea	Prospective cohort	15 exposed 313 unexposed	BP > 140/90 mmHg on two separate occasions with at least 1 + proteinuria on a random urine screen after the 20th week of pregnancy	two or more inter-proximal sites with CAL ≥ 4 mm that were not on the same tooth	at 21–24 weeks of gestation	The association was much stronger in women with both obesity and periodontitis	15.94 (3.31–76.71)
28	(Khalighinejad et al., 2017)	USA	Case–control	50 cases 50 controls	A systolic blood pressure ≥ 140 mm HG or a diastolic pressure ≥ 90 mm HG and proteinuria > 300 mg/24 h developed after the 20^th^ week of gestation	The presence of 4 or more teeth with 1 or more sites with PD ≥ 4 mm and with clinical attachment loss ≥ 3 mm at the same site	Before delivery	Apical periodontitis was significantly more prevalent in the experimental group	2.23 (1.92–6.88)
29	(Lafaurie et al., 2018)	Colombia	Case–control	76 cases 304 controls	not mentioned	the patients were classified according to the presence of periodontal pockets (code 3: periodontal pockets of 4-5 mm or code 4: periodontal pockets > 5 mm)	during the first week after birth	Periodontal pockets presence was not associated with preeclampsia	5.46 (1.84- 16.1)
30	(Jaiman et al., 2018)	India	Case–control	15 cases 15 controls	preeclampsia as the appearance of a diastolic blood pressure ≥ 90 mmHg mercury measured at two different occasions at least 4 h apart in combination with proteinuria (≥ 300 mg/24 h or + 1 dipstick) developing after a gestational age of 20 weeks in a previously normotensive woman	According to the criteria of Löe and Silness	24 h before delivery	The preeclamptic women were associated with significantly higher periodontitis and lower fetal birth weight than normotensive women	Periodontitis in case group was 93.3% and in control group was 33.3% (p < 0.05)

*BP* blood pressure, *DBP* diastolic blood pressure, *SBP* systolic blood pressure, *GW* gestational week, *PD* pocket depth, *CAL* clinical attachment loss, *OHI* oral hygiene index, *GI* gingival index, *BOP* bleeding on probing, *GCF* gingival crevicular fluid

The sample size ranged from 40 participants (Varshney & Gautam, 2014) to 1240 subjects (Desai et al., 2015). Seven studies reported no evidence of an association between periodontitis and preeclampsia (Chaparro et al., 2013; Hirano et al., 2012; Horton et al., 2010; Khalighinejad et al., 2017; Lafaurie et al., 2018; Lohsoonthorn et al., 2009; Pattanashetti et al., 2013; Shetty et al., 2009; Taghzouti et al., 2012), while the remaining studies reported a positive association. Studies which had controlled for factors such as age, weight, smoking or occupation were reported(Boggess et al., 2003; Canakci et al., 2004, 2007; Cota et al., 2006; Desai et al., 2015; Ha et al., 2011, 2014; Hirano et al., 2012; Horton et al., 2010; Khader et al., 2006; Kumar et al., 2013; Kunnen et al., 2007; Lafaurie et al., 2018; Lee et al., 2016; Lohsoonthorn et al., 2009; Moura da Silva et al., 2012; Politano et al., 2011; Pralhad et al., 2013; Sayar et al., 2011; Shetty et al., 2009; Siqueira et al., 2008; Soucy-Giguère et al., 2016; Taghzouti et al., 2012).

### Risk of Bias of Included Studies

Newcastle Ottawa Scale was used to evaluate the quality of evidence of these reports. Two reviewers marked the scores for each paper based on the tool provided by the Scale. Nine studies (Ha et al., 2011, 2014; Jaiman et al., 2018; Khader et al., 2006; Khalighinejad et al., 2017; Lee et al., 2016; Moura da Silva et al., 2012; Pattanashetti et al., 2013; Soucy-Giguère et al., 2016) obtained the maximum score in Selection outcome while fourteen studies were marked with maximum score in the Comparability outcome and none of the studies could achieve ultimately 3 marks in the Exposure outcome. Table 2 describes the evaluation of risk of bias for this review.

**Table 2:** Risk of bias in included studies based on Newcastle–Ottawa scale

Study, year	Selection (Max 4*)	Comparability (Max 2*)	Exposure (Max 3*)	Risk of bias
Boggess 2003	***	*	**	High
Canakci 2004	***	**	**	Moderate
Contreras 2006	***	*	**	High
Cota 2006	***	*	**	High
Khader 2006	****	**	**	Moderate
Kunnen 2007	***	**	**	Moderate
Canakci 2007	***	**	**	Moderate
Siqueira 2008	***	*	**	High
Lohsoonthorn 2009	***	**	**	Moderate
Horton 2010	***	*	**	High
Shetty 2010	***	**	**	Moderate
Politano 2011	***	**	**	Moderate
Ha 2011	****	**	**	Moderate
Sayar 2011	***	**	**	Moderate
Taghzouti 2012	***	**	**	Moderate
Chaparro 2012	***	*	**	High
Hirano 2012	***	*	**	High
Kumar 2012	***	*	**	High
da Silva 2012	****	**	**	Moderate
Pralhad 2012	***	**	**	Moderate
Yaghini 2012	***	**	**	Moderate
Pattanashetti 2013	****	**	**	Moderate
Ha 2014	****	**	**	Moderate
Varshney 2014	***	**	**	Moderate
Desai 2015	***	**	**	Moderate
Soucy-Giguere 2015	****	**	***	Low
Lee 2016	****	*	**	Moderate
Khalighinejad 2017	****	**	**	Moderate
Lafaurie 2018	***	*	**	High
Jaiman 2018	****	**	**	Moderate

### Synthesis of Results

**Fig. 2:**
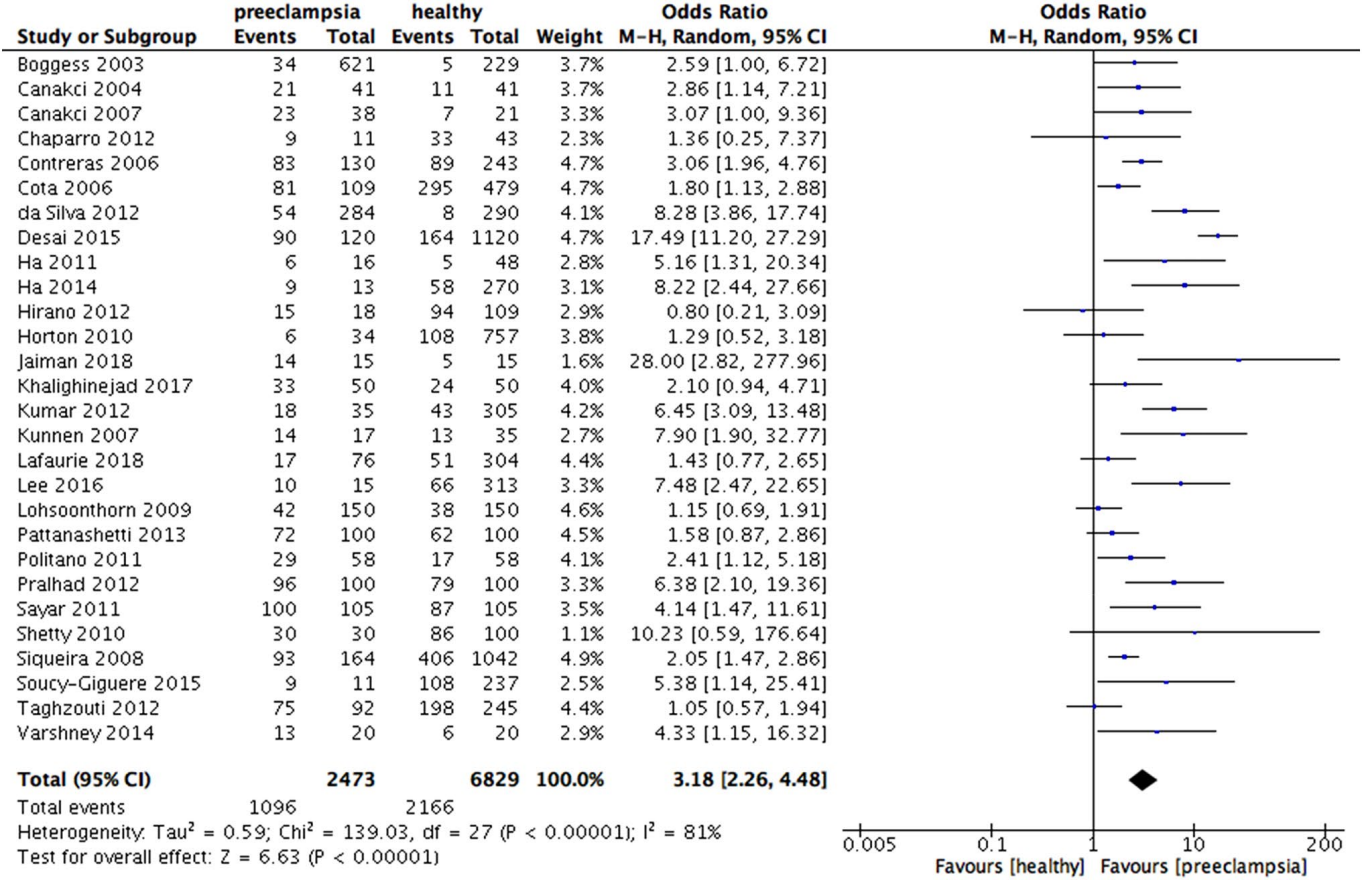
Forest plot for the association between periodontitis and preeclampsia

The results of the meta-analysis showed that periodontitis was associated with increased risk for preeclampsia (OR 3.18, 95% CI 2.26 – 4.48, p < 0·00,001; Fig. 2). The heterogeneity was high (I^2^ = 81%, p < 0.00001) revealing a significant variation among studies.

### Subgroup Analysis

**Fig. 3:**
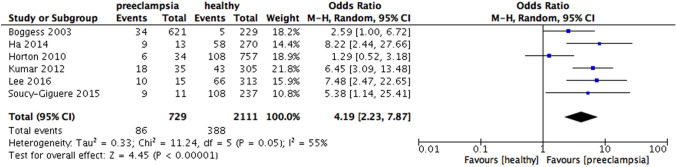
Forest plot for the subgroup analysis according to the type of study design (cohort study)

**Fig. 4:**
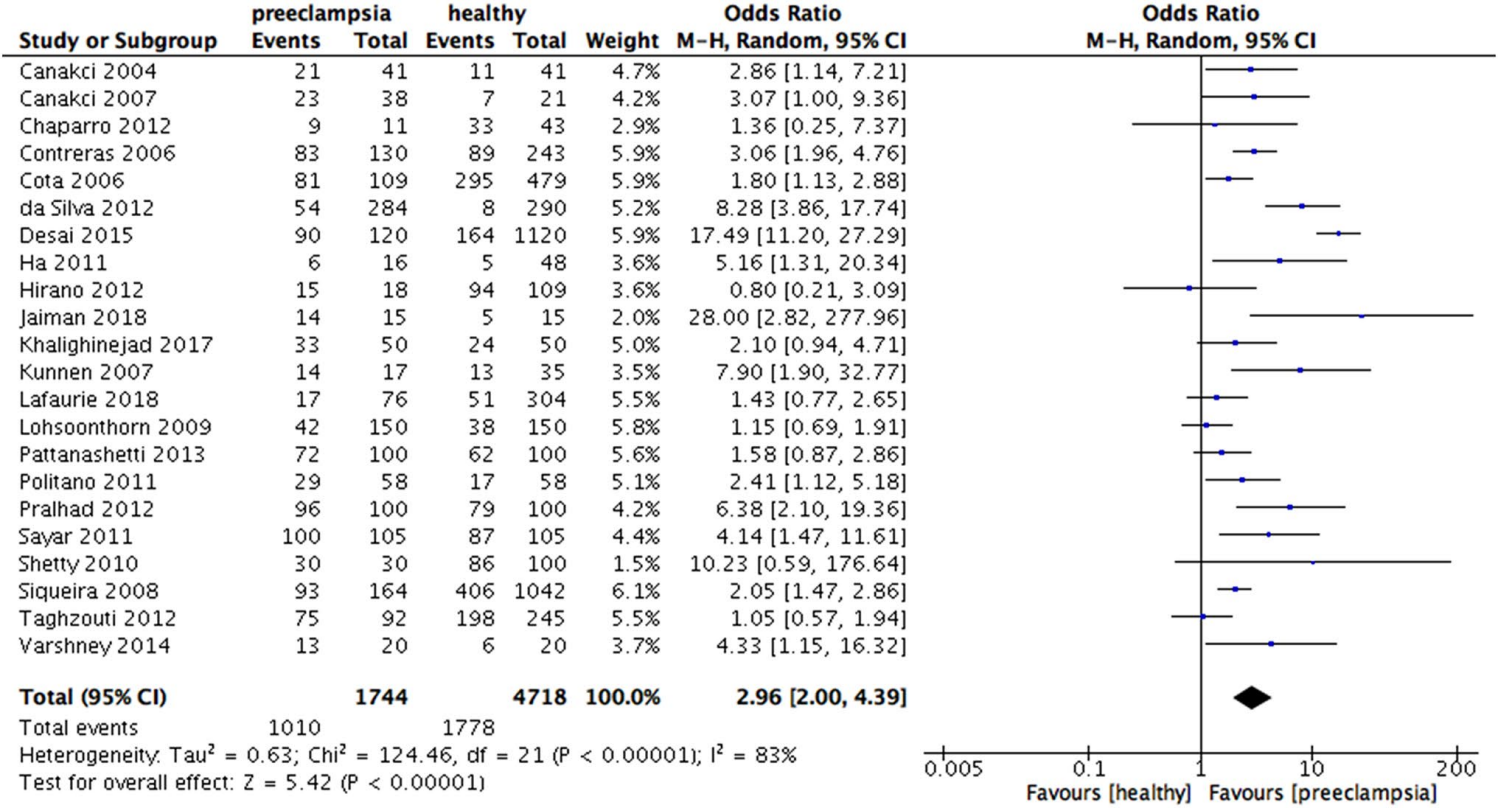
Forest plot for the subgroup analysis according to the type of study design (case–control study)

According to the study type, the results revealed the increased risk of preeclampsia in periodontitis patients in the cohort studies (OR 4.19, 95% CI 2.23 – 7.87, p < 0.00001; Fig. 3) and in case–control studies (OR 2.96, 95% CI 2.00 – 4.39, p < 0.00001; Fig. 4). Heterogeneity was moderate for cohort (I^2^ = 55%, p = 0.05) but high for case–control (I^2^ = 83%, p < 0.00001).

**Fig. 5:**
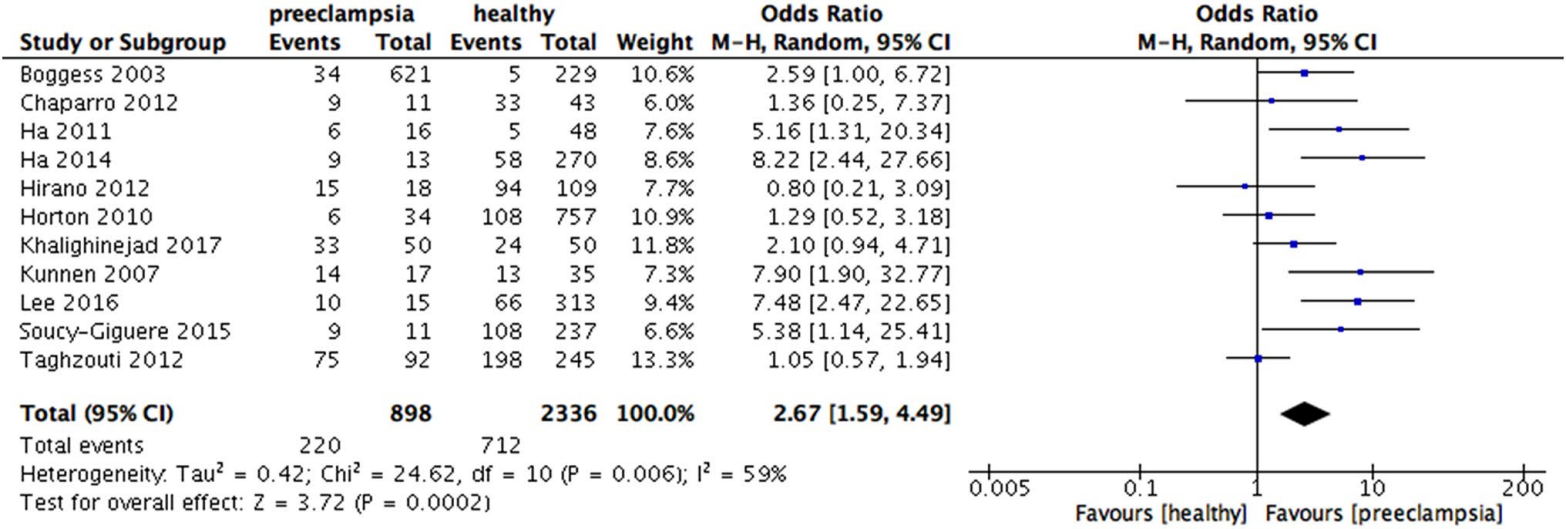
Forest plot for the subgroup analysis according to the national income (high income countries)

**Fig. 6:**
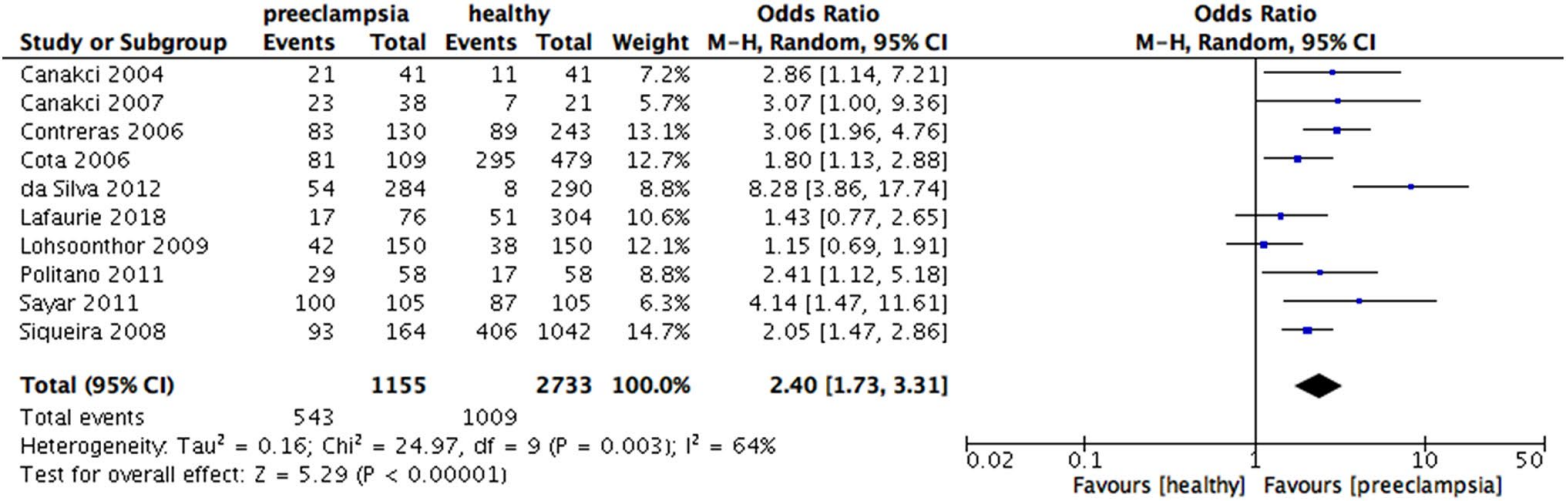
Forest plot for the subgroup analysis according to the national income (upper middle-income countries)

**Fig. 7:**
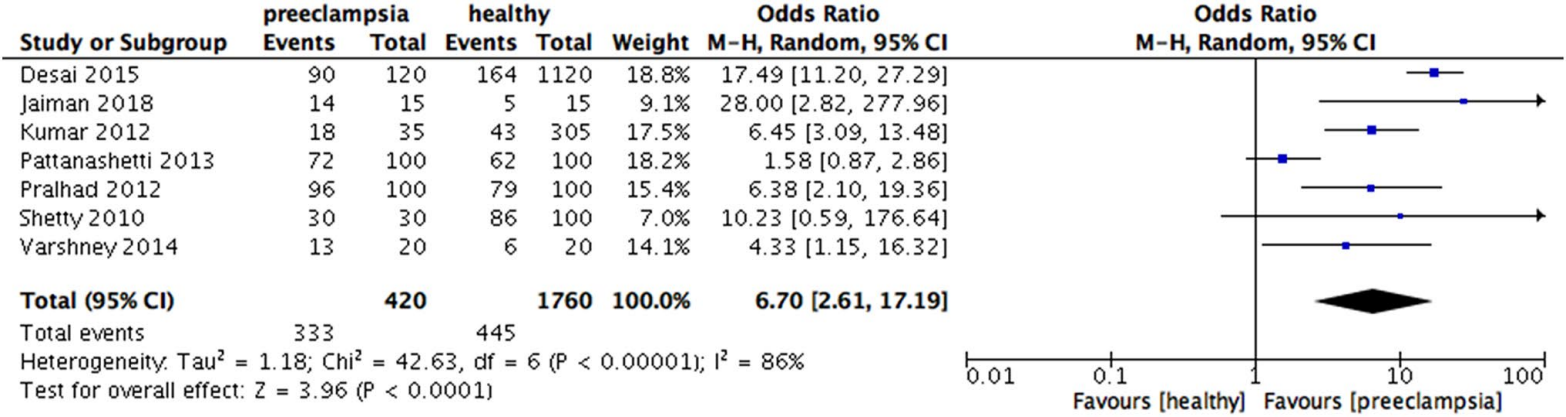
Forest plot for the subgroup analysis according to the national income (lower middle- income countries)

**Fig. 8:**
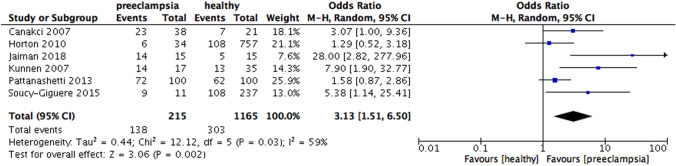
Forest plot for the subgroup analysis according to the definition of periodontitis (PD alone)

When analyzing according to the national income, increased risk of preeclampsia were found in periodontitis group in high-income countries (OR 2.67, 95% CI 1.59 – 4.49, p = 0.0002; Fig. 5), upper middle-income countries (OR 2.40, 95% CI 1.73 – 3.31, p < 0.00001; Fig. 6) and especially lower-middle income countries (OR 6.7, 95% CI 2.61 – 17.19, p < 0.0001; Fig. 7). Heterogeneity in the group of high-income, upper middle-income and lower middle-income countries were moderate (I^2^ = 59%, p = 0.006; I^2^ = 64%, p = 0.003; I^2^ = 86%, p < 0.00001, respectively) (Fig. 8).

**Fig. 9:**
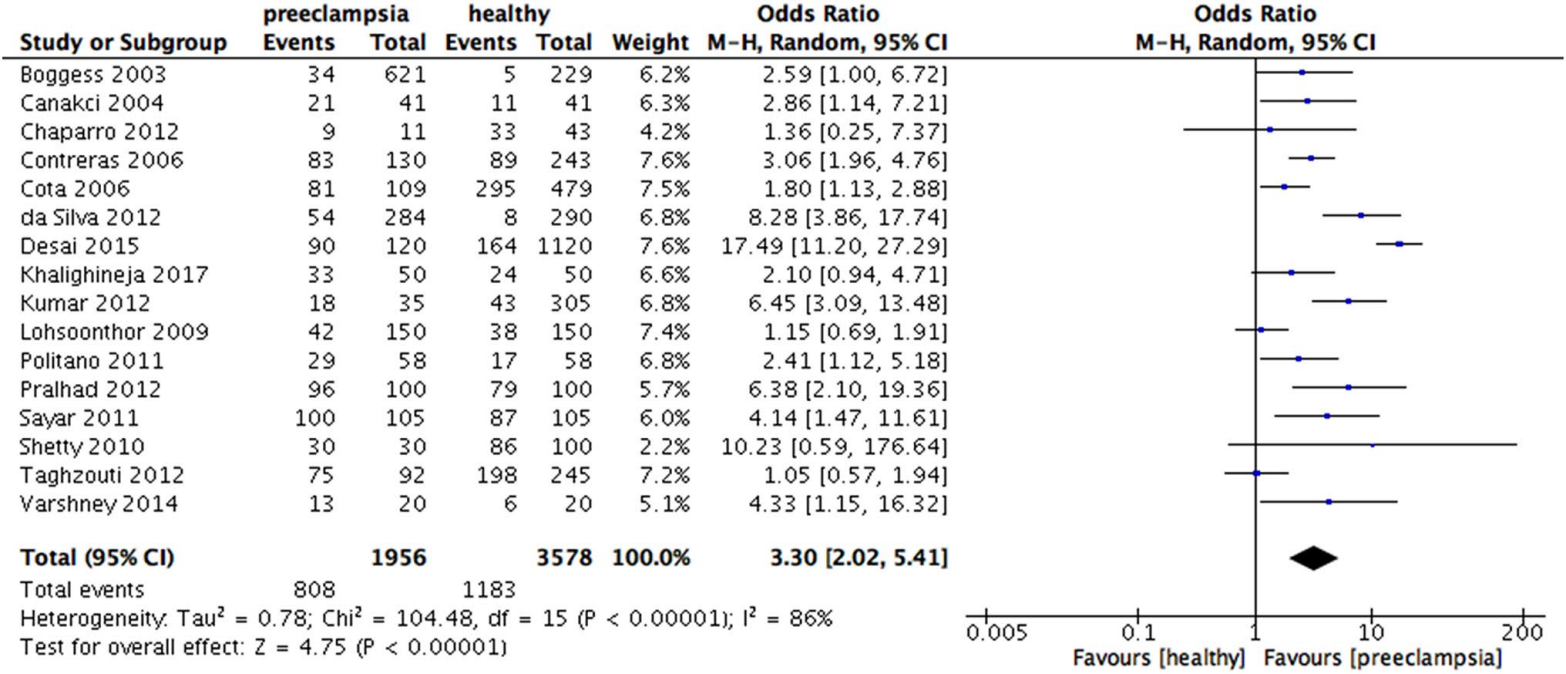
Forest plot for the subgroup analysis according to the definition of periodontitis (PD and CAL)

When the results were analyzed according to the definition of periodontitis, an increased risk of preeclampsia was observed in all subgroups, including PD only (OR 3.13, 95% CI 1.51 – 6.50, p = 0.002), CAL and PD (OR 3.30, 95% CI 2.02 – 5.41, p < 0.00001), CAL alone (OR 2.74, 95% CI 1.50 – 5.01, p = 0.001). Heterogeneity was moderate for the subgroups in which periodontitis was defined by PD alone (I^2^ = 59%, p = 0.03) and CAL alone (I^2^ = 66%, p = 0.01), while significantly high heterogeneity was found in the subgroup which periodontitis was defined by CAL and PD ( I^2^ = 86%, p < 0.00001) (Fig. 9).

**Fig. 10:**
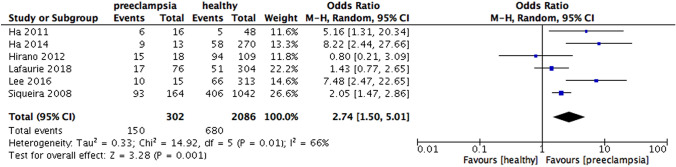
Forest plot for the subgroup analysis according to the definition of periodontitis (CAL alone)

**Fig. 11:**
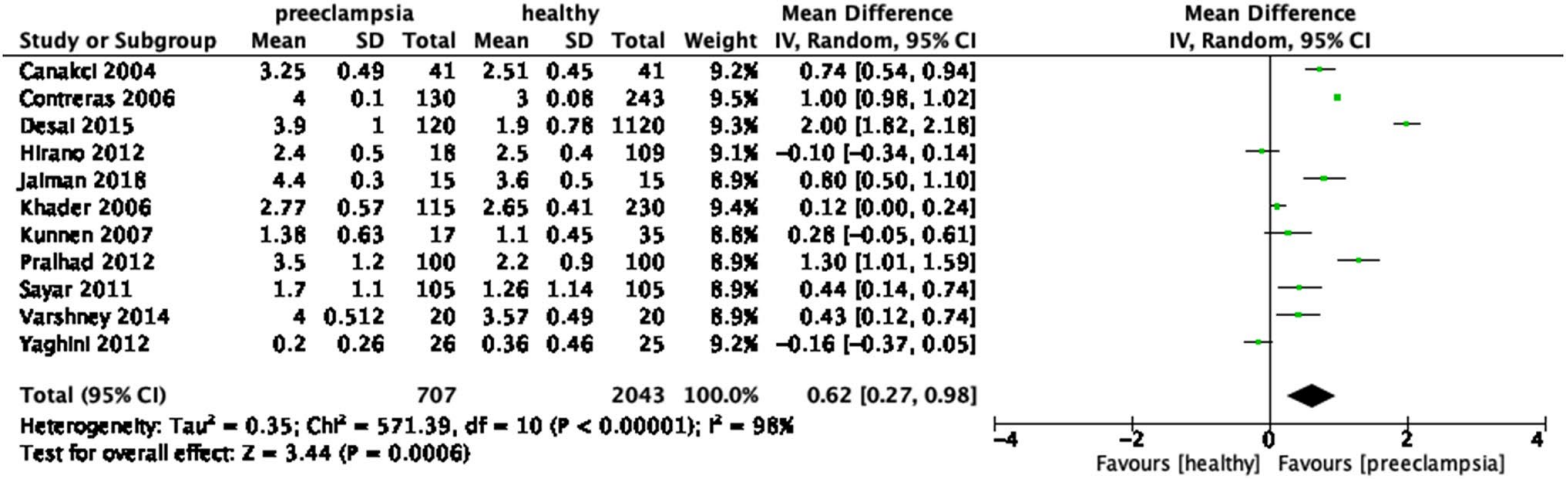
Forest plot for the subgroup analysis of mean CAL between preeclamptic and healthy groups

**Fig. 12:**
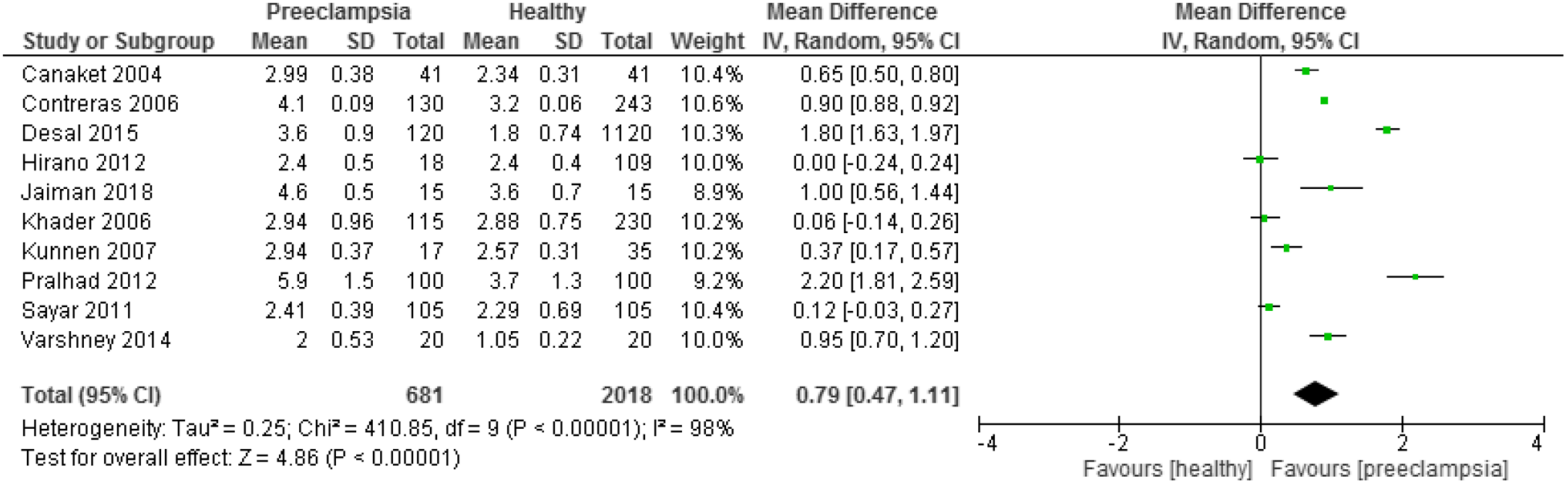
Forest plot for the subgroup analysis of mean PD between preeclamptic and healthy groups

When analyzing the periodontal condition between both groups, mean CAL was statistically higher in the preeclamptic patients than in the healthy group (MD = 0.62, 95% CI 0.27 – 0.98, p = 0.0006). Likewise, the preeclamptic group had a statistically higher mean PD compared to healthy group (MD = 0.79, 95% CI -0.47 – 1.11, p < 0.00001). The heterogeneity was 98% in both subgroup analysis, (I^2^ = 98%, p < 0.00001) (Figs. 10, 11 and 12).

### Publication Bias

**Fig. 13:**
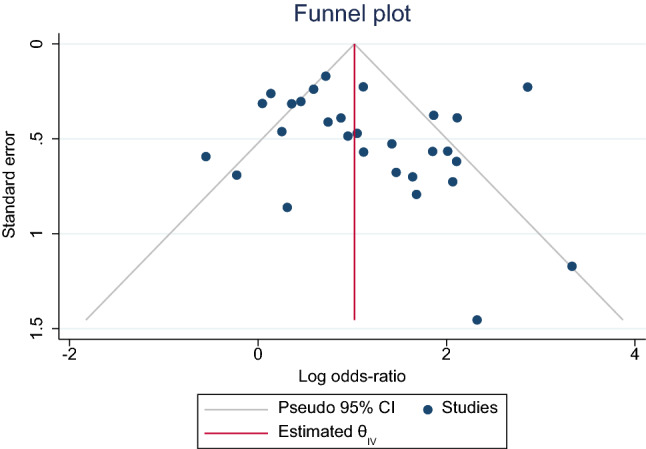
Funnel plot for the association between periodontitis and preeclampsia

The funnel plot for the association between periodontitis and preeclampsia revealed the symmetry (Fig. 13). No publication bias was found.

## Discussion

The aim of this meta-analysis was to re-evaluate the potential association between preeclampsia and periodontitis. The results confirm that periodontitis is a risk factor for preeclampsia, which was similar to the finding of a meta-analysis in 2013 by Sgolastra et al. (Sgolastra et al., 2013). Our review has fifteen additional studies with three more cohort studies considerably increasing the sample size and hence generating more robust effect sizes and significance levels.

By stratifying according to study designs, periodontitis and preeclampsia showed significant associations in both case–control and cohort studies, whereas Sgolastra et al. (Sgolastra et al., 2013) could not report the statistical significance in the subgroup analysis of cohort studies (OR 2.2, 95% CI 0.66 – 7.36, p = 0.2). As a review of cohort studies provides higher level of evidence compared to case–control studies (Guyatt et al., 2000) our data considerably strengthens the evidence of a positive association between preeclampsia and periodontitis. Moreover, the heterogeneity in an analysis of cohort studies in our study (I^2^ = 55%, p = 0.05) was substantially lower than in a study by Sgolastra et al. (I^2^ = 89%, p = 0.0001), again strengthening the reliability of our results (Sgolastra et al., 2013).

When analyzed according to the definition of periodontitis, three subgroup analysis with studies defining periodontitis by PD alone, CAL and PD and CAL alone showed statistically significant differences, whereas the previous meta-analysis showed only significance with a subgroup analyzing periodontitis by CAL and PD. This could be explained by the number of studies included in our review was more than in the previous analysis, thus, providing a more comprehensive finding. However, according to the most recent case definition developed by the Centre for Disease Control and Prevention in partnership with the American Academy of Periodontology, the diagnostic criteria of periodontitis is at least 2 interproximal sites with the minimum of attachment loss of 3 mm and at least 2 interproximal sites with the minimum pocket depth of 4 mm (not on the same tooth) or one site with pocket depth ≥ 5 mm (Eke et al., 2012). Moreover, pregnant women who were preeclamptic had higher mean CAL and PD, however, the heterogeneity in both subgroup analysis was high, indicating significant variations among these studies in each subgroup. The high heterogeneity reported could result from the difference in the periodontal probes used in the dental examination in each study.

Several mechanisms have been proposed for the link between periodontitis and preeclampsia. Higher levels of some periodontal pathogens such as *P.gingivalis* and *F. nucleatum* were found in placenta of patients with preeclampsia (Barak et al., 2007). Moreover, inflammatory responses including the shifting of Th2 toward Th1, increasing oxidative stress, anti-angiogenic proteins, vascular endothelial growth factor receptor 1 and complement C5a could potentially enhance the development of preeclampsia (Nourollahpour Shiadeh et al., 2017). Ananth et al. has reported the association between intrauterine growth restriction and maternal periodontitis (Ananth et al., 2018). Since severe and early onset preeclampsia were associated significantly with fetal growth restriction, this could contribute to the mechanism underlying the association between preeclampsia and periodontitis (Odegård et al., 2000). Furthermore, the mechanisms might be a reflection of dietary patterns. Recently, some evidence has indicated that pathogenesis of preeclampsia involves maternal gut microbiota, specifically, high-fiber diet which promote short chain fatty acid production and are associated with reduced risk of preeclampsia (Hu et al., 2019). Similarly, high-fiber foods such as fruit and grains have been linked to the reduction of the progression of periodontal disease, suggesting the role of dietary intake in the potential relationship between preeclampsia and periodontal disease (Hu et al., 2019; Schwartz et al., 2012). However, future studies are required to elucidate these hypotheses.

When analyzing the association between periodontitis and preeclampsia according to national income, this review revealed the significant difference in the subgroup analysis of high-income and upper-middle-income countries (OR = 2.67 and OR = 2.40, respectively). Moreover, the subgroup analysis with lower-middle-income countries, which generated an Odds ratio of 6.70, indicated the considerable significance in the relationship between periodontitis and preeclampsia in this specific country group. The high heterogeneity was observed in the subgroup of lower-middle-income countries which implies the variation between included countries. Countries were categorized as lower-middle-income using national gross income per capita, thereby being subject to variation even within each individual country. In other words, using national income as a proxy may result in this variation and therefore, suggested the individual level approach for future studies to tackle this issue. Moreover, lower middle-income countries have poorer oral health condition compared to upper-middle and high-income country groups, which may indicate the inequalities in oral health care (Bastani et al., 2021; Watt & Sheiham, 1999). Inequalities can stem from unjust provision of services or inappropriate access and become more pronounced by the fact that most dental treatment is funded by out-of-pocket payments (Listl et al., 2015).

Socioeconomic inequalities in access to oral health care accounted for 60% in lower-middle-income countries (Hosseinpoor et al., 2012). Therefore, improving access to oral health services for pregnant women in lower-middle-income countries is notably important to lessen the risk of having preeclampsia. Additionally, the allocation of resources for oral care might need more investigations and strategic management to eliminate the disparities in oral health care (Arevalo & Tomar 2019). Social and cultural determinants include biological, behavioral, cultural, social and political aspects should also be focused to thoroughly eradicate the inequalities(Patrick et al., 2006).

We used Newcastle Ottawa Scale to evaluate the risk of bias and found twenty studies with moderate risk of bias and ten remaining studies with a high risk of bias. Exposure bias in this study was due to the non-response rate which was not described clearly in these studies. No publication bias was detected. Sensitivity analysis resulted in no change to the finding of the study.

The strength of this systematic review and meta-analysis includes the large sample size of 9650 subjects. Six cohort studies comprising 2840 subjects were analyzed and revealed the statistically significant difference. Because systematic reviews of prospective cohort studies generate more reliable evidence, our study provided an updated systematic review and meta-analysis and confirmed the association between periodontitis and preeclampsia (Hillier et al., 2011). Furthermore, by stratifying into subgroup analysis of national income, our review has pointed out the association between these two diseases differed according to economical inequalities, thus, providing recommendation for health policy improvement. Pregnant women in low socioeconomical areas should be given access to oral healthcare services and encouraged to have their periodontal health checked and treated during pregnancy to potentially lower the risk of preeclampsia and other pregnancy complications. Jeffcoat et al. reported non-surgical periodontal therapy could significantly reduce the medical costs for pregnant women by 73.7% (Jeffcoat et al., 2014). We acknowledged few limitations in our study. Firstly, the heterogeneity of the overall analysis for the association between periodontitis and preeclampsia was high, pointing out the variations among studies included. The synthesis of cohort and case–control studies in our review may explain for this high heterogeneity. Secondly, the general consensus in the definition and diagnosis of periodontitis was not clear enough which could influence the results of our meta-analysis. Additionally, because the intraoral examinations were conducted at different time points, the diagnosis of periodontitis may be impacted. Deteriorated periodontium was observed more in the third trimester compared to the second trimester and overall periodontal health was improved postpartum (González-Jaranay et al., 2017; Kashetty et al., 2018). Thereby, future clinical studies should consider the time point of seven days of the elivery when conducting periodontal examinationsand confirm our results. Moreover, because our analysis was based on national income, new research with individual-level data of socioeconomic factors is recommended for a more informative conclusion.

## Conclusions and Implications

This meta-analysis confirms previous findings of an association between periodontitis and preeclampsia. However, this study includes fifteen more recent publications, which resulted in a larger effect size of the association, specifically, for lower-middle-income countries in comparison to high and upper-middle-income countries. Our results warrant future studies to investigate the mechanisms of this association and whether targeted interventions to prevent or treat periodontitis preconception or during pregnancy can lead to better pregnancy outcomes.

## Data Availability

Data will be available upon request.

## Abbreviations

PD: Pocket depth
CAL: Clinical attachment loss
